# It is time to consider the climate crisis in haematology

**DOI:** 10.46989/001c.133524

**Published:** 2025-03-27

**Authors:** Robin Noel, Aude Charbonnier, Bérénice Schell, Arthur Dony, Charles Toulemonde, François Eisinger, Olivier Decaux, Joanna Lotocka, Edith Julia, Alya Perthus, Mathilde Seguin, Aurélie Cabannes-Hamy, Pierre Sujobert, Laurie Marrauld, Caroline Besson

**Affiliations:** 1 Hematology Institut Paoli-Calmettes, France; 2 Haematological Biology Centre Hospitalier Universitaire Henri-Mondor, France; 3 Hematology Centre Hospitalier Métropole Savoie, France; 4 Hematology Hospices Civils de Lyon, France; 5 Research Institut Paoli-Calmettes, France; 6 Hematology Centre Hospitalier Universitaire de Rennes; 7 Hematology Centre Hospitalier Universitaire de Rennes, France; 8 Hematology Assistance Publique – Hôpitaux de Paris, France; 9 Hematology Centre Hospitalier de Versailles, France; 10 Hematological Biology Hospices Civils de Lyon, France; 11 University of Rennes École des Hautes Études en Santé Publique, France

**Keywords:** Haematology, public health, climate change, clinical trials

Climate crisis (CC), pollution and destruction of biodiversity represent a major threat to present and future societies. Global pollution is the single largest cause of environmental deaths, with approximately 9 million premature deaths estimated per year.^1^ As stated by the World Health Organization, ‘recent research attributes 37% of heat-related deaths to human-induced climate change” and ’conservatively projects 250,000 additional yearly deaths by the 2030s due to climate change impacts’.^2^ The Intergovernmental Panel on Climate Change (IPCC) publishes comprehensive reports on the potential impacts of on society’s health and well-being, possible future challenges and risks,^3^ along with recommendations for mitigation and adaptation; the Lancet Countdown monitors the evolving health profile of climate change, providing an independent assessment of the delivery of commitments made by governments worldwide under the Paris Agreement.^4,5^

The healthcare system faces a dilemma with its own significant impact on the environment, particularly in wealthy countries, and the increasing climate-related morbidity and mortality. According to The Shift Project, the French healthcare system is responsible for over 8% of the national carbon footprint (40 to 61 million tons of CO_2_ equivalent (MtCO_2_e), i.e. 6.6 to 10%; **Figure 1**), with roughly 50% of these greenhouse gas (GHG) emissions coming from the procurement of drugs and medical devices,^6^ findings which align with other studies and confirm the significant carbon impact of the healthcare systems in developed countries.^7,8^

**Figure 1.**
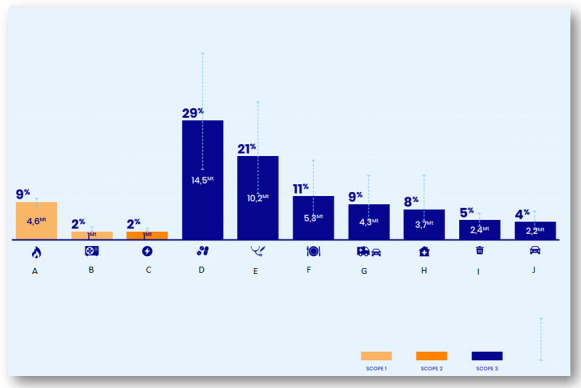
Distribution of GHG emissions from the French health sector (MTCO_2_e: Metric Tons of carbon dioxide equivalents CO_2_ - *Courtesy of the ShiftProject*): A: Energy (heating, water heating, etc.) B: Medical gases consumption and air conditioning C: Electricity D: Procurement: drug purchase E: Procurement: medical equipment purchase F: Food supply G: Patients and visitors transportation H: Manufacturing of durable goods (buildings, IT system, heavy medical devices, etc.) I: Waste and services J: Staff commuting

Many believe that in order to ensure humanity’s protection, healthcare should be addressed and assessed like other sectors of the economy in light of the ecological transition. As stated in a United Nations Charter itself, ‘our present actions shouldn’t jeopardize everyone’s future as a matter of transgenerational and global responsibility’.^9^ Others might argue that medical care should always prevail over other considerations - as some sort of ‘sanctuary’ - and not be taken into account when mitigating GHG emissions and pollution, an argument that could also apply to other sectors such as transport, agriculture or defense. Nevertheless, reducing GHG emissions is a matter of complying with carbon-neutral goals by 2050, and the early decarbonization of healthcare will therefore strengthen its resilience in a changing world.

It is time to tackle this vast subject of evaluating and reducing healthcare GHG emissions. Others and we believe that just as with any goods and services, the environmental footprint of medicines and medical devices must be assessed and disclosed to medical providers, patients and all citizens. Measuring GHG emissions is an emerging topic in medicine. Little is known about the impact of treatments in hematology, and very few studies have been published in other medical fields (dialysis, critical care, etc.) to assess more sustainable treatment strategies.^10–18^

While innovation is one of the pillars of hematology, there is currently limited data on the impact of new drugs or therapeutic strategies (CART-cells, tyrosine kinase inhibitors, bispecific antibodies, etc.) on GHG emissions.^19^ In practice, we mainly assess the benefit/risk ratio of our treatments based on the uniqueness of each patient according to survival rates and safety profiles. We should estimate the environmental impact of our therapeutic strategies and promote research to achieve the same goals regarding survival and quality of life with lower GHG emissions. For example, the use of stop-and-start strategy versus continuous treatment could be expanded beyond chronic myeloid leukemia. Tyrosine kinase inhibitors have successfully allowed exploration of stop-and start treatments without impairing the survival of patients, while reducing costs and resources.^20^ This approach has also recently been implemented in chronic lymphoid leukemia, although in other chronic diseases such as multiple myeloma the standard largely remains continuous treatment. It is of utmost importance to question such an approach, and that sequential treatments should be systematically investigated in clinical trials.

Moreover, clinical trials should be transparent about their global GHG production and commit to using sustainable methods.^21–23^ The Low Carbon Clinical Trials Group^22^ provides methods and guidance for reducing the carbon footprint of clinical trials. Emissions from clinical trial units, as well as travel by staff and patients, are identified as the primary sources of GHG emissions. In this context, remote enrollment and tele monitoring could have a significant impact. Follow-up exams are often numerous and may be reduced particularly in asymptomatic patients with favorable chronic conditions, such as follicular lymphoma for instance. Additionally, using synthetic control arms based on data from recent trials can reduce the number of patients needed for enrollment.^24^ Moreover, they should also consider their environmental impact as an output and gradually change their model to test therapeutic modalities with lower emissions such as shorter treatment durations, with a global approach to health.

As hematologists, simple changes to our daily routine can have significant positive impacts. For example, increasing the number of online consultations,^25–27^ cutting down on the amount of unnecessary diagnostics and routine monitoring exams, or favoring the administration of treatment orally over IV.^28^ Moreover, promoting more sustainable and equitable medical meeting attendance via hybrid conferences, for example, can have a strong impact.^29^ Finally, in the future, leading expert opinion groups for each neoplasm should also consider incorporating environmental impact into their decision-making when issuing routine guidelines, as data become available.

Education on this subject is fundamental and should be incorporated into all levels of the medical curriculum to ensure future physicians promote sustainable medical practices. Seeing as this topic extends beyond the medical community and touches all members of society, as physicians, we have a responsibility to raise public awareness, enabling people to take part in the discussion and transition process.

Finally, to achieve a greater purpose we must advocate for more drastic measures on a larger scale. Policy makers and drug agencies - such as the FDA and EMA - should implement impact assessments of drugs and therapeutic strategies for both present and future patients. We need long-term and global assessments of the benefit/risk ratio, otherwise, future generations might not benefit at all from our innovations. Governmental regulatory authorities could implement environmental criteria within the research field, thus fostering eco-responsible research projects and guidelines on how to mitigate the CC impact.

In conclusion, the CC is also a dire health emergency. Our medical obligations are not only to offer the best possible patient care based on current knowledge, but from a broader public health perspective, to ensure people are able to maintain a high quality of health going forward, while incorporating sustainable medical practices in our decision-making.^30^ This would provide an opportunity to create deeper meaning and responsibility within hematology, and to motivate future generations of health care providers. If the medical community chooses to ignore the warning signals expressed by other scientific communities, we might fail our primary duty to our patients’ health, jeopardize that of future generations, and breach the guidance of good medical practice.

## Authors’ Contribution

Conceptualization: Robin Noel (Equal), Aude Charbonnier (Equal), Caroline Besson (Equal). Writing – original draft: Robin Noel, Aude Charbonnier (Equal), François Eisinger (Equal), Caroline Besson (Equal). Writing – review & editing: Bérénice Schell (Equal), Arthur Dony (Equal), Charles Toulemonde (Equal), Olivier Decaux (Equal), Joanna Lotocka (Equal), Edith Julia (Equal), Alya Perthus (Equal), Mathilde Seguin (Equal), Aurélie Cabannes-Hamy (Equal), Pierre Sujobert (Equal), Laurie Marrauld (Equal).

## Competing of Interest - COPE

Authors have no competing interests to disclose for this article.
